# Fabrication of Chitosan Hybrid Particles with Shrimp Shell Protein Hydrolysate–EGCG Conjugates for Antioxidant Protection in Lipid Systems

**DOI:** 10.3390/antiox15091163

**Published:** 2026-09-12

**Authors:** Akanksha R Gautam, Soottawat Benjakul, Avtar Singh, Ravindran Muthukumarasamy, Nilesh Nirmal, Saleh Al-Ghamdi, Mohammad Fikry

**Affiliations:** 1 International Centre of Excellence in Seafood Science and Innovation (ICE-SSI), Faculty of Agro-Industry, Prince of Songkla University, Hat Yai 90110, Songkhla, Thailand; 2 Department of Food and Nutrition, Kyung Hee University, Seoul 02447, Republic of Korea; 3 Department of Pharmaceutical Chemistry, Faculty of Pharmacy and Health Sciences, Royal College of Medicine Perak, Universiti Kuala Lumpur, Ipoh 30450, Perak, Malaysia; 4 Institute of Nutrition, Mahidol University, Salaya, Phutthamonthon, Nakhon Pathom 73170, Thailand; 5 Department of Agricultural Engineering, King Saud University, P.O. Box 2460, Riyadh 11451, Saudi Arabia; 6 Date Palm Research Center of Excellence, King Faisal University, Al-Ahsa 31982, Saudi Arabia

**Keywords:** hybrid particles, shrimp shell, thermal stability, particle size, antioxidant, lipid oxidation

## Abstract

Shrimp shell–derived chitosan and protein hydrolysate–epigallocatechin gallate conjugates (SEC) were used to fabricate hybrid particles (C-SEC) at mass ratios of 1:1, 1:5, and 1:10 using an alkali precipitation method. SEC incorporation significantly enhanced the antioxidant capacity of chitosan particles (*p* < 0.05). Among the formulations, C-SEC prepared at a ratio of 1:5 (C–SEC–1:5) exhibited the highest antioxidant activities, including DPPH (14.55 mM TE/g) or ABTS (9.80 mM TE/g), or hydroxyl (70.29%) radical scavenging activities, ferric reducing antioxidant power (10.59 mM TE/g), and metal chelating activity (6.37 mM EE/g). The particle size was markedly reduced from 738 nm for chitosan particles (CP) to 112 nm following incorporation of SEC, indicating the formation of finely dispersed hybrid particles. ^1^H NMR analysis confirmed successful molecular integration of SEC into the chitosan matrix through non-covalent interactions. Thermal analyses revealed altered degradation and transition behavior of the hybrid particles. TGA showed that the principal degradation stages shifted to higher temperatures, whereas DSC showed an increase in transition enthalpy from 370.74 to 2449.60 J/g. SEM and TEM images revealed that the C–SEC–1:5 formed porous, interconnected particle networks with rough surfaces. The selected hybrid particles also showed superior inhibition of Cu^2+^–induced lecithin liposome oxidation and β-carotene bleaching compared with CP, demonstrating concentration-dependent protection against lipid oxidation. Overall, these findings indicate the potential of C–SEC–1:5 as an antioxidant-based functional ingredient and bioactive delivery vehicle in food and nutraceutical formulations.

## 1. Introduction

The increasing consumer demand for natural, sustainable, and health-promoting food ingredients has stimulated extensive research into biopolymer-based delivery systems capable of enhancing the stability and functionality of bioactive compounds [1]. Among these systems, hybrid particles formed through the combination of proteins, polyphenols, and polysaccharides have emerged as promising carriers for bioactive compounds due to their ability to overcome the individual limitations of each component. Such hybrid systems have demonstrated significant potential in food, nutraceutical, and pharmaceutical applications, particularly for improving the stability, bioavailability, and controlled release of sensitive bioactive compounds [2].

Marine processing waste represents an underutilized source of various functional additives or bioactive compounds, such as chitin and proteins from shrimp shell [3]. During shrimp shell processing, deproteinization separates protein from the shell matrix, while the remaining chitin can subsequently be converted into chitosan through deacetylation. Chitosan is known to possess excellent properties including biocompatibility, biodegradability, film-forming ability, antimicrobial activity, and mucoadhesive properties due to its molecular composition [4]. The presence of amino groups in the glucosamine units of chitosan enables electrostatic interactions with negatively charged molecules, facilitating the formation of particles, complexes, and delivery systems. For instance, Rajabimashhadi et al. [5] developed chitosan microparticles through ionic gelation using tripolyphosphate (TPP), where electrostatic interactions between the positively charged amino groups of chitosan and negatively charged phosphate groups of TPP promoted microparticle formation. Despite these advantages, chitosan-based systems may exhibit limitations under a neutral or alkaline environment, which limit their solubility and thus their functional properties. Therefore, the modification of chitosan via conjugation with other biomolecules such as proteins, polyphenols, polysaccharides, etc., could overcome these limitations. For example, Liang et al. [6] fabricated casein phospho-peptide–chitosan composite hybrid particles through electrostatic interactions between chitosan and negatively charged groups of casein phospho-peptides. The resulting hybrid particles showed improved structural stability and quercetin encapsulation efficiency. Thus, the combination of chitosan with value-added products from shrimp processing waste such as protein recovered during chitin production could be used as a component of a hybrid system.

Shrimp shell protein hydrolysates (SPH), which consist of peptides with diverse molecular weights and amino acid compositions, many of which exhibit beneficial biological activities, including antioxidant, antihypertensive, and metal-chelating properties, have been produced in our previous study [3,7]. Furthermore, their activities and functional properties have been enhanced by conjugating SPHs with epigallocatechin gallate (EGCG). The combination of protein–polyphenol conjugates with polysaccharides offers additional opportunities for designing hybrid particles with tailored structural and functional characteristics [8]. Although chitosan–polyphenol and chitosan–protein composite systems have been widely investigated, the use of SPH–EGCG conjugates as a functional component for the fabrication of chitosan–based hybrid particles remains limited. Therefore, integrating the SPH–EGCG conjugate (SEC) with chitosan represents a relatively unexplored approach for developing hybrid particles that combine the functional properties of chitosan, protein-derived peptides, and EGCG. Interactions between chitosan and protein-based materials are primarily governed by electrostatic attraction, hydrogen bonding, and hydrophobic interactions, which can promote the formation of stable complexes and particulate systems [9]. The incorporation of the SPH–EGCG conjugate (SEC) into chitosan matrices may generate hybrid particles exhibiting improved colloidal stability, enhanced antioxidant properties, and desirable physicochemical characteristics [10].

Therefore, the present study aimed to fabricate hybrid particles based on chitosan and SEC. The resulting particles were characterized for their physicochemical, morphological, structural, thermal, and antioxidant properties. Furthermore, the antioxidant potential of the obtained particles was also determined in an in vitro system using lecithin liposome oxidation and β-carotene–linoleic acid bleaching assay models.

## 2. Materials and Methods

### 2.1. Raw Materials and Chemicals

Pacific white shrimp (*Litopenaeus vannamei*) shells were obtained from Sea Wealth Frozen Food (Songkhla, Thailand). Food-grade alcalase (EC 3.4.21.62; 124 U/mL) derived from *Bacillus licheniformis* was purchased from Siam Victory Chemicals Co., Ltd. (Bangkok, Thailand). Epigallocatechin gallate (EGCG) was ordered from Xi’an Julong Bio-Tech Co., Ltd. (Xi’an, China). Low-molecular-weight chitosan (MW 50–190 kDa; degree of deacetylation 75–85%) was obtained from Sigma-Aldrich (St. Louis, MO, USA). All chemicals and reagents used in this study were of analytical grade.

### 2.2. Preparation of Shrimp Shell Protein Hydrolysate–EGCG Conjugate (SEC) 

The SEC was prepared following the previously optimized conditions of Gautam et al. [11]. Shrimp shell protein hydrolysate (SPH; 2%) was dissolved in distilled water and mixed with an ascorbic acid/hydrogen peroxide redox pair (2%, *v*/*v*) at 27 °C for 2 h under dark conditions to generate reactive radicals. Subsequently, EGCG (0.5%, *w*/*v*) was added to the activated SPH solution, and the mixture was stirred at 27 °C for 24 h in the dark to facilitate covalent conjugation. The resulting SEC was dialyzed (500 Da molecular weight cutoff) against distilled water at 4 °C for 24 h to remove unbound EGCG and was then subjected to freeze-drying for further use. The conjugation efficiency was 91.73%.

### 2.3. Preparation of Chitosan–SEC (C-SEC) Hybrid Particles

Chitosan solution (1%, *w*/*v*) was prepared by dissolving chitosan in 1% (*v*/*v*) acetic acid under continuous magnetic stirring at room temperature. The pH of the solution was adjusted to 5.5 using 1 M NaOH. Separately, SEC solution (1%, *w*/*v*) was prepared in 10% (*v*/*v*) ethanol and stirred until completely dispersed. The SEC solution was mixed with the chitosan solution at chitosan–to–SEC mass ratios of 1:1, 1:5, and 1:10 under continuous stirring at room temperature. The ratios were selected to provide progressively increasing proportions of SEC relative to chitosan, representing low, intermediate, and high SEC incorporation levels, respectively. Hybrid particle formation was induced by the dropwise addition of 1 M NaOH (20 mL) under constant stirring. This resulting in the formation of an alkaline suspension with a pH of approximately 12.5, which was subsequently subjected to ultrasonication, using a probe sonicator (Sonics, Model VC750, Sonics & Materials, Inc., Newtown, CT, USA; high-intensity power: 750 W and operating frequency: 20 kHz ± 50 Hz) at 60% amplitude for 10 min in pulse mode to enhance particle dispersion and minimize aggregation. The ultrasonication condition was selected based on the preliminary study. The formed hybrid particles were collected by centrifugation. The collected pellet was repeatedly washed with distilled water followed by centrifugation until the pH of the washing water approached neutral. The washed particles were then freeze-dried and stored for subsequent characterization and analysis. Chitosan particles (CP) were also prepared without the SEC using the same method.

### 2.4. Characterization of CP and C-SEC Hybrid Particles

#### Antioxidant Activities

The antioxidant activities of the samples (200 ppm in water) were determined by hydroxyl radical scavenging activity (HRSA), DPPH radical scavenging activity (DPPH-RSA), ABTS radical scavenging activity (ABTS-RSA), ferric reducing antioxidant power (FRAP), and metal chelating activity (MCA) according to the methods of Siddhuraju et al. [12] with slight modifications.

For HRSA, 0.5 mL sample solution was mixed with 0.5 mL of 10 mM phosphate buffer (pH 7.4), containing 2.8 mM deoxyribose, 2.8 mM H_2_O_2_, 25 μM FeCl_3_, and 100 μM EDTA. After 1 h of reaction at 37 °C, the absorbance of each mixture was measured at 532 nm using a UV-1601 spectrophotometer (Shimadzu, Kyoto, Japan). The HRSA was calculated as follows:

$$HRSA\left(\%\right)=\left(1-\frac{A_{532}^{sample}}{A_{532}^{control}}\right)\times 100$$

$A_{532}^{sample}$ = absorbance at 532 nm in the presence of sample.
$A_{532}^{control}$ = absorbance at 532 nm in the absence of sample (control).

The DPPH-RSA was measured by combining 1.5 mL of the sample solution with 1.5 mL of 0.1 mM DPPH solution in 95% ethanol. This mixture was then incubated for 30 min at room temperature in the dark. After incubation, the absorbance was read at 517 nm. A control was prepared under identical conditions, substituting the sample with distilled water. The activity was quantified using a Trolox standard curve (0–60 µM) and expressed as μM Trolox equivalents (TE)/g of solids.

For ABTS-RSA, a stock solution of 14.8 mM ABTS and 5.2 mM potassium persulfate were used. Equal volumes of the stock solutions were mixed and allowed to react for 12 h in the dark at room temperature, to prepare the working solution. This solution was then diluted with methanol to achieve an absorbance of 1.1 ± 0.02 at 734 nm. The activity was determined using a Trolox standard curve, ranging from 0 to 600 μM, and expressed as μM TE/g of solids.

The FRAP assay was performed using a freshly prepared reagent composed of 10 mM 2,4,6-tris(2-pyridyl)-s-triazine (TPTZ) in 40 mM HCl, 20 mM FeCl_3_·6H_2_O, and 300 mM acetate buffer at pH 3.6 (in a ratio of 1:1:10, *v*/*v*/*v*). This reagent (2.85 mL) was incubated at 37 °C for 30 min. Then, 150 μL of the sample was added, and the mixture was left in the dark at room temperature for 30 min. The absorbance was measured at 593 nm. The activity was calculated based on a Trolox standard curve (0–600 μM) and expressed as μM TE/g of solids.

MCA was assessed by mixing 200 μL of the sample with 800 μL of distilled water, followed by the addition of 0.1 mL of 2 mM FeCl_2_ and 0.2 mL of 5 mM ferrozine. The reaction mixture was allowed to stand at room temperature for 20 min, and the absorbance was measured at 562 nm. An EDTA standard curve was used (0–30 μM) to express chelating activity as μM EDTA equivalents (EE)/g of solids. A blank was prepared in the same manner, with distilled water substituted for the sample.

The C–SEC ratio exhibiting the optimal antioxidant activities was selected for further study.

### 2.5. Particle Size

The mean particle size of CP and the selected C-SEC was measured using a laser diffraction particle size analyzer (Model LS 230, Beckman Coulter, Fullerton, CA, USA) [13]. 

### 2.6. Thermogravimetric Analysis (TGA)

TGA was performed under a nitrogen (N_2_) atmosphere using a Mettler Toledo TGA/DSC3+ thermogravimetric analyzer (Mettler-Toledo, Greifensee, Switzerland). Approximately 5–10 mg of each dried sample was placed in a sample pan and heated from 25 to 500 °C at a constant heating rate of 10 °C/min. The thermogravimetric data were analyzed using STARe thermal analysis software (v16.40a).

### 2.7. Differential Scanning Calorimetry (DSC)

DSC analysis of CP and selected C-SEC was performed using a DSC8500 (PerkinElmer, Shelton, CT, USA) following the method described by Mittal et al. [14]. The samples were scanned between −80 °C and 380 °C at a heating rate of 5 °C/min under an N_2_ atmosphere. The normalized heat flows as a function of temperature were recorded and the corresponding enthalpy changes were determined from the DSC thermograms.

### 2.8. Nuclear Magnetic Resonance (NMR)

CP and C–SEC–1:5 were characterized using proton nuclear magnetic resonance (^1^H–NMR) with the aid of a 500 MHz Fourier transform NMR spectrometer (Unity Inova, Varian, Germany).

### 2.9. Scanning Electron Microscopy (SEM)

The microstructures of CP and C–SEC–1:5 were analyzed using a scanning electron microscope (SEM) (Quanta 400, FEI, Eindhoven, The Netherlands). The samples were mounted on individual bronze stubs and sputter-coated with a gold layer (Sputter Coater SPI-Module, West Chester, PA, USA).

### 2.10. Transmission Electron Microscopy (TEM)

TEM was used to characterize the morphology of the prepared samples using a TALOS F200i transmission electron microscope (Thermo Fisher Scientific, Waltham, MA, USA) operated at an accelerating voltage of 100 kV. Water dispersions of the hybrid materials were cast onto carbon-coated copper grids and allowed to air-dry. The dried grids were then examined by TEM.

### 2.11. Antioxidant Potential of Different Particles at Various Levels

#### 2.11.1. Lecithin Liposome Oxidation Assay

The antioxidant activity of the samples was evaluated using a lecithin liposome oxidation model according to the method of Sharma et al. [15] with slight modifications. Lecithin was dispersed in deionized water at a concentration of 8 mg/mL and sonicated for 30 min using a sonication bath to obtain a homogeneous liposome suspension. Subsequently, the sample was mixed with 15 mL of lecithin liposome suspension for 2 min to achieve final concentrations of 100, 200, and 500 mg/L. Lipid oxidation was initiated by adding 20 µL of 0.15 M cupric acetate solution. The reaction mixtures were incubated in the dark at 37 °C with continuous shaking at 120 rpm. Butylated hydroxyanisole (BHA, 100 mg/L) was used as a positive control, while a liposome system containing cupric acetate without an antioxidant served as the negative control. The extent of lipid oxidation was monitored by measuring thiobarbituric acid reactive substances (TBARSs) at 0, 6, 12, 24, 36, and 48 h of incubation. Malondialdehyde (MDA) standards (0–3 mg/L) were used to generate a calibration curve for quantification. TBARS values were expressed as mg MDA equivalents/mL of liposome suspension.

#### 2.11.2. β-Carotene–Linoleic Acid Bleaching Assay

The antioxidant activity of the samples was evaluated using the β-carotene–linoleic acid bleaching assay [15]. Briefly, β-carotene (10 mg) was dissolved in 10 mL of chloroform. An aliquot (0.2 mL) of the β-carotene solution was transferred to a round-bottom flask containing linoleic acid (20 mg) and Tween 40 (200 mg). The chloroform was completely removed under a stream of nitrogen gas. Subsequently, 50 mL of oxygenated deionized water was added, and the mixture was vigorously homogenized to form a stable emulsion. CP and C-SEC-1:5 were added to the emulsion to obtain final concentrations of 100, 200, and 500 mg/L. Butylated hydroxyanisole (BHA, 100 mg/L) was used as the positive control, while deionized water was used as the negative control. The reaction mixtures were incubated at 50 °C in the dark for 390 min. The absorbance was measured at 470 nm at 15 min intervals throughout the incubation period using a spectrophotometer. The antioxidant activity was evaluated based on the ability of the samples to inhibit β-carotene bleaching. A slower decrease in absorbance at 470 nm indicated a greater capacity to prevent linoleic acid oxidation and protect β-carotene from oxidative degradation.

### 2.12. Statistical Analysis

The experiments were conducted in triplicate, and a completely randomized design (CRD) was used. The data were subjected to analysis of variance (ANOVA), followed by the comparison of means using Duncan’s multiple range test. Statistical analysis was carried out using the Statistical Package for the Social Sciences (SPSS 11.0 for Windows, SPSS Inc., Chicago, IL, USA). Statistical significance was accepted at *p* < 0.05.

## 3. Results and Discussion

### 3.1. Effect of Various Ratios of Chitosan and SEC on the Antioxidant Activities of Hybrid Particles

The antioxidant potential of the prepared C-SEC hybrid particles was evaluated using five antioxidant assays, including HRSA, DPPH–RSA, ABTS–RSA, FRAP, and MCA. As shown in Table 1, incorporation of the SEC markedly enhanced the antioxidant activities of hybrid particles as compared with chitosan particles prepared without the SEC (CP). The maximum HRSA inhibition was obtained when chitosan was combined with the SEC at a ratio of 1:5 (70.29%), followed by 1:1 (60.19%) and 1:10 (47.28%). The lowest inhibition was noticed for CPs (23.70%). Similarly, DPPH–RSA was the maximum at a ratio of 1:5 (14.55 mM TE/g) followed by 1:10 (13.18 mM TE/g) and 1:1 (12.88 mM TE/g) (*p* < 0.05). Likewise, the ABTS assay demonstrated significantly improved radical scavenging activity for the hybrid particles relative to CP (4.93 mM TE/g), in which particles prepared at a 1:5 ratio (9.80 mM TE/g) showed the strongest antioxidant response as compared to the other ratios such as 1:10 (7.97 mM TE/g) and 1:1 (7.28 mM TE/g) (*p* < 0.05).

The reducing capacity of the hybrid particles was assessed by the FRAP assay. C–SEC particles prepared at a ratio of 1:5 had the highest ferric-reducing capacity (10.57 mM TE/g) compared with the 1:1 and 1:10 ratios (7.65 and 7.05 mM TE/g, respectively). This indicates an enhanced electron transfer capability for reducing ferric (Fe^3+^) ions to ferrous (Fe^2+^) ions. MCA also followed a similar trend, with C–SEC (1:5) (6.37 mM TE/g) displaying the greatest chelation capacity, followed by 1:10 (6.20 mM TE/g), 1:1 (4.25 mM TE/g) and CPs (3.75 mM TE/g). The enhanced chelating ability is particularly significant because transition metal ions, especially Fe^2+^, catalyze the formation of highly reactive oxygen species through Fenton-type reactions, which could lead to the oxidation of lipids and proteins. Therefore, effective sequestration of these metal ions contributes to preventing oxidative damage by suppressing radical generation [16].

The improvement in antioxidant activity observed for the hybrid particles can be attributed to the synergistic effects of chitosan and SPH/EGCG components of the SEC. Chitosan contributes to the antioxidant activity through its free amino and hydroxyl groups, which can donate hydrogen atoms or electrons to quench free radicals. In addition, the amino groups of chitosan can chelate transition metal ions, thereby inhibiting metal-catalyzed oxidative reactions and reducing the generation of reactive oxygen species [4]. EGCG is a well-established natural polyphenolic antioxidant containing multiple phenolic hydroxyl groups capable of donating hydrogen atoms and electrons to neutralize free radicals while stabilizing the resulting phenoxyl radicals through resonance [17]. Simultaneously, SPH contributes antioxidant peptides containing amino acid residues such as histidine, tyrosine, methionine, cysteine, and hydrophobic amino acids, which participate in radical scavenging, electron donation, and chelation of transition metal [3]. The combination of these bioactive components within the chitosan matrix provides multiple complementary antioxidant mechanisms, resulting in significantly enhanced overall antioxidant performance [17]. A similar synergistic effect was reported by Liang et al. [6], who found that quercetin-loaded casein phosphopeptide/chitosan particles exhibited 95.57% DPPH radical-scavenging activity, which was higher than that of casein phosphopeptide–quercetin particles (72.14%) and chitosan–quercetin particles (88.47%). The superior antioxidant activity observed for C–SEC–1:5 compared with C–SEC–1:1 and C–SEC–1:10 may be associated with a favorable balance between SEC incorporation and the chitosan matrix. The incorporation of the SEC provides additional antioxidant-active components, particularly the phenolic hydroxyl groups of EGCG and bioactive peptide residues derived from SPH, which can contribute to radical scavenging, electron transfer, and metal chelation [17]. At the 1:1 ratio, the lower proportion of SEC may result in a lower availability of these antioxidant-active groups. In contrast, the higher SEC proportion at the 1:10 ratio did not further increase antioxidant activity, which may be related to changes in intermolecular interactions and particle organization that could affect the accessibility of antioxidant-active groups. Thus, the higher activity of C–SEC–1:5 reflects a favorable balance. Compared with CP, C–SEC–1:5 increased HRSA, DPPH–RSA, ABTS–RSA, FRAP, and MCA by approximately 196.6%, 83.7%, 98.6%, 181.7%, and 69.8%, respectively, highlighting the substantial improvement in antioxidant functionality following SEC incorporation. Overall, the consistent enhancement observed in DPPH–RSA, HRSA, ABTS–RSA, FRAP, and MCA demonstrates that incorporation of the SEC conjugate substantially improved the antioxidant functionality of chitosan hybrid particles through multiple antioxidant mechanisms, including radical scavenging, electron transfer, reducing power, and transition metal chelation. Based on these comprehensive antioxidant evaluations, C–SEC–1:5 was the optimal formulation among the tested hybrid particle systems. Thus, C–SEC–1:5 was selected for subsequent physicochemical characterization and application studies.

### 3.2. Characterization of Chitosan Particles (CP) and Selected Hybrid Particles (C–SEC–1:5)

#### 3.2.1. Particle Size

CP exhibited an average particle size of 738 nm, whereas C–SEC–1:5 showed a significantly smaller diameter of 112 nm, which is approximately 84.8% smaller than CPs. The particle size of the ultrasound-treated samples was also smaller than that of the corresponding non-ultrasound-treated samples (CP: 987.4 nm and C–SEC–1:5; 476.3 nm). Ultrasonication at an amplitude higher than 60% resulted in the disintegration of particles, which led to the formation of coagulum material rather than a stable particle system. Therefore, an amplitude of 60% for 10 min was selected for particle preparation. The larger size of CP can be primarily attributed to the alkali-induced precipitation and aggregation mechanism of chitosan. Upon addition of NaOH, deprotonation of the protonated amino groups (−NH_3_^+^) reduces electrostatic repulsion and decreases chitosan solubility, leading to rapid polymer chain association and phase separation. Consequently, multiple chitosan chains associate to form gel nuclei that subsequently grow into larger particles during the alkalization process [18]. The resulting particle structure is further stabilized by intermolecular hydrogen bonding and chain entanglement among chitosan molecules, contributing to the formation of mechanically stable particles [19]. In contrast, when SEC was incorporated into chitosan, it restricted particle growth, resulting in the formation of uniformly dispersed hybrid particles during the alkalization process. In general, particle size is likely driven by multiple non-covalent interactions, including hydrogen bonding, electrostatic interactions, and hydrophobic interactions between chitosan and the SEC [20]. EGCG possesses multiple phenolic hydroxyl groups capable of forming hydrogen bonds with the amino and hydroxyl groups of chitosan, while simultaneously interacting with functional groups of SPH. Similarly, Bourouis et al. [10] demonstrated that incorporation of EGCG into protein–chitosan complexes promoted structural reorganization through hydrogen bonding, electrostatic interactions, and hydrophobic interactions, leading to the formation of more compact and stable ternary complexes. These intermolecular interactions likely promoted the formation of a more compact and structurally organized network, thereby preventing excessive particle aggregation. Moreover, the peptide component of the SEC may provide steric stabilization, further inhibiting particle coalescence during hybrid particle formation. Zhang et al. [21] reported that the physical processing-induced rearrangement and self-assembly of soy peptide aggregates resulted in the formation of smaller and more homogeneous particles with the resulting soy peptide nanoparticles exhibiting particle sizes of 318.75–595.30 nm. Ethanol, used as the solvent for the SEC, likely influenced the self-assembly behavior of the polymeric system by promoting controlled nucleation and limiting uncontrolled particle growth, contributing to the controlled assembly behavior of the hybrid system by influencing intermolecular interactions during particle formation [22]. Doan and Ghosh [23] reported that ethanol-induced desolvation could modify protein–protein interactions and influence particle formation by altering solvent polarity and reducing protein solubility, thereby affecting particle structure and size distribution. They observed a reduction in particle size from 500 ± 10 nm to approximately 350–580 nm following ethanol-induced desolvation and subsequent homogenization. The formation of hybrid particles with an average diameter of 112 nm is particularly advantageous for functional applications due to the enhanced surface area, which increases the availability of surface-active functional groups for interaction with surrounding media. Overall, these findings demonstrate that incorporation of SEC effectively altered the self-assembly behavior of chitosan during particle formation, producing stable hybrid particles with enhanced physicochemical characteristics that are favorable for subsequent functional and delivery applications.

#### 3.2.2. Thermogravimetric Analysis (TGA)

**Figure 1:**
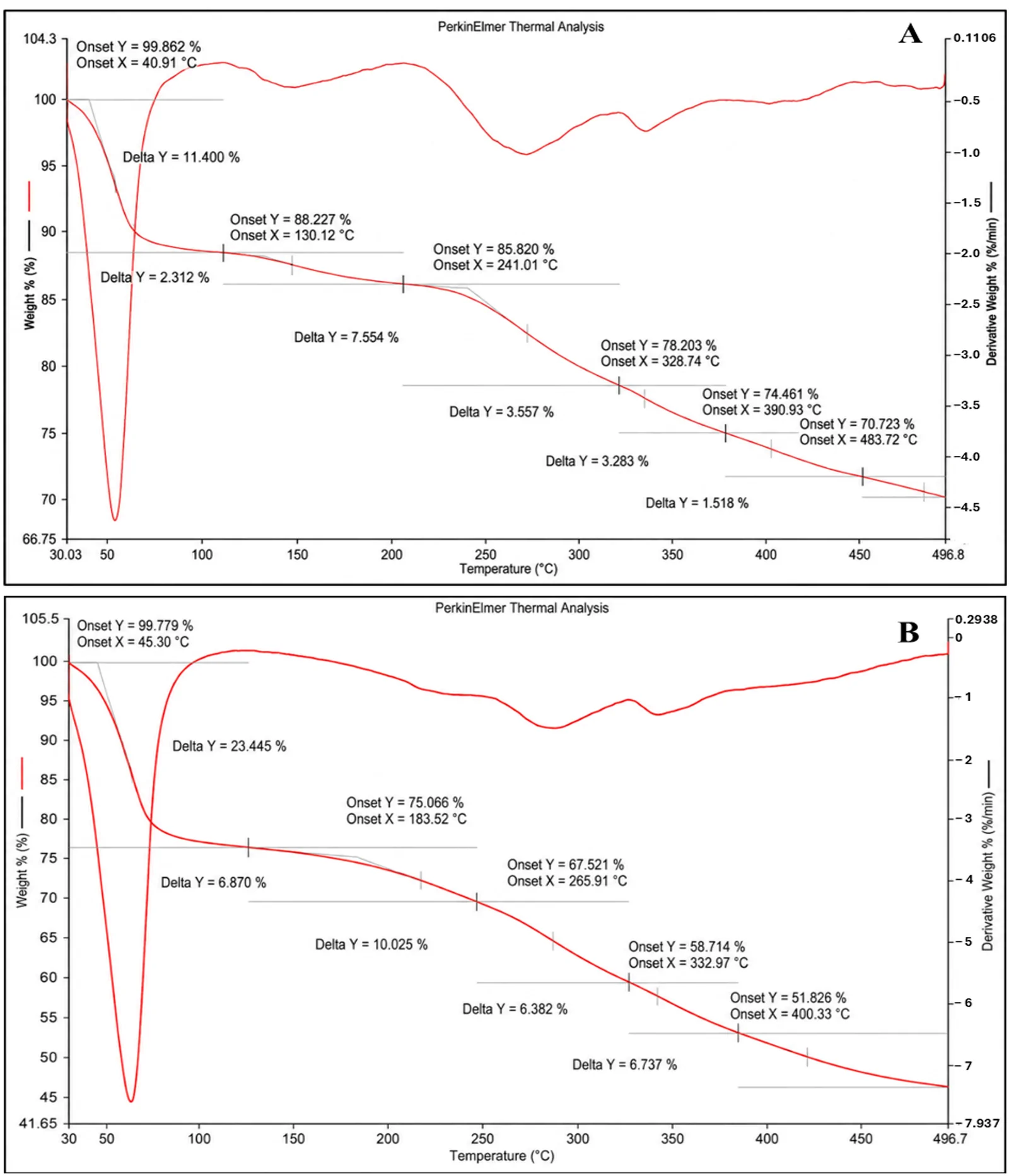
The thermogravimetric analysis (TGA) of chitosan particles (CP) (**A**) and chitosan–shrimp shell protein hydrolysate–EGCG conjugate (C–SEC) hybrid particles (**B**).

The TGA was performed to investigate the thermal stability and degradation behavior of CP and C–SEC–1:5 hybrid particles (Figure 1A and Figure 1B, respectively). The thermograms exhibited multi-stage thermal degradation patterns, indicating structural differences between CP and C–SEC–1:5. The TGA curve of CP showed an initial weight loss below 100 °C, with an onset temperature of approximately 40.91 °C and a mass loss of about 11.40%. Similar thermal behavior has been reported by Ahmed et al. [24], in which chitosan hybrid particles showed the initial weight loss at lower temperatures was associated with the removal of free and bound water molecules. This was more likely due to the hydrophilic characteristics of chitosan. A second minor degradation stage was observed around 130.12 °C, with a weight loss of approximately 2.31%, which might be correlated with the removal of residual bound water and unstable functional groups. The major degradation stage was noticed at 241.01, 328.74, and 390.93 °C, exhibiting successive weight losses of 7.55%, 3.56%, and 3.28%, respectively. Georgieva et al. [25] also reported that the main thermal degradation of chitosan occurred above approximately 240 °C due to the cleavage of glycosidic bonds, depolymerization of the polysaccharide chains, and subsequent decomposition of the polymer structure. A further small degradation stage observed near 483.72 °C with a weight loss of 1.52% was related to decomposition of the residual carbonaceous char [26]. The comparatively higher residual mass at elevated temperature indicated moderate thermal stability of the chitosan particles. 

In contrast, C-SEC-1:5 particles exhibited a distinct thermal degradation profile. The initial weight loss started at approximately 45.30 °C with a relatively higher weight loss (23.45%) than that of CP. This could be attributed to the evaporation of moisture together with thermally labile components of the SEC. Similar initial weight loss behavior has been observed in modified pea protein-based hybrid particles, where moisture and volatile compounds contributed to the first weight loss stage during TGA analysis [27]. Furthermore, the presence of EGCG may influence the thermal behavior of the hybrid particles through interactions between phenolic hydroxyl groups and the chitosan matrix, as reported for chitosan–polyphenol composite systems [28]. The second weight loss stage observed around 183.52 °C showed a weight loss of approximately 6.87%, which could be associated with thermal decomposition of peptide fractions from SPH as well as degradation of EGCG constituents. The principal degradation regions occurred at 265.91, 332.97, and 400.33 °C, with major weight losses of 10.03%, 6.38%, and 6.74%, respectively. Compared to the CP, major degradation stages of the hybrid particles shifted toward higher temperatures, suggesting modified thermal behavior and improved resistance to intermediate thermal degradation. This phenomenon may be attributed to intermolecular interactions, including hydrogen bonding and electrostatic interactions among chitosan, SPH, and EGCG molecules [29]. Similarly, Zhang et al. [13] reported that incorporation of pea protein isolate into chitosan hybrid particles enhanced thermal stability due to the formation of hydrogen bonding and electrostatic interactions between chitosan and protein molecules, which improved the structural integrity of the hybrid system. Additionally, Bourouis et al. [10] demonstrated that incorporation of EGCG into soy protein isolate-chitosan complexes promoted the formation of ternary complexes through hydrogen bonding, electrostatic interactions, and hydrophobic interactions. These molecular interactions induced structural reorganization and improved the thermal stability of the hybrid system, supporting the enhanced thermal resistance observed in the present C–SEC particles. Nevertheless, the hybrid particles exhibited greater overall mass loss and lower residual mass at 500 °C compared to CP, likely due to the presence of thermally sensitive peptide and polyphenolic constituents [30]. Overall, the TGA results demonstrated that incorporation of the SEC significantly altered the thermal degradation characteristics of chitosan particles, confirming successful hybrid particle formation. Improved thermal resistance and modified degradation behavior may contribute to enhanced stability of encapsulated bioactive compounds during processing and storage.

#### 3.2.3. Differential Scanning Calorimetry (DSC)

**Figure 2:**
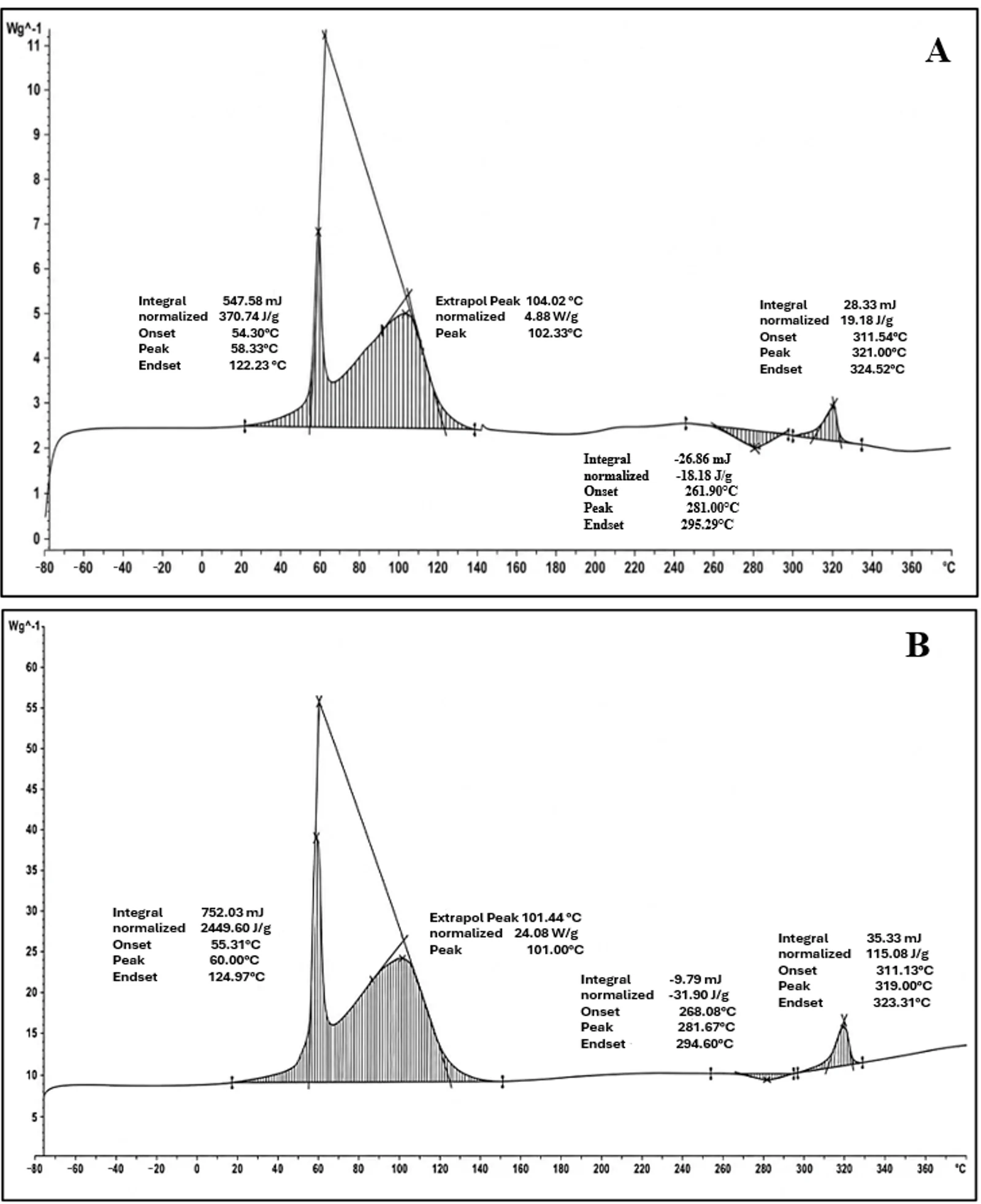
The differential scanning calorimetry (DSC) analysis of chitosan particles (CP) (**A**) and chitosan–shrimp shell protein hydrolysate–EGCG conjugate (C–SEC) hybrid particles (**B**).

DSC thermograms of CP and C-SEC-1:5 particles are presented in Figure 2A and Figure 2B, respectively. The samples were initially equilibrated at −80 °C for 1 min and subsequently heated from −80 °C to 380 °C at a heating rate of 5 °C/min. The DSC thermogram of CP exhibited a broad endothermic transition between 54.30 °C and 122.23 °C with a peak temperature at 58.33 °C and an enthalpy value of 370.74 J/g. This thermal event was mainly attributed to the evaporation of physically adsorbed and bound water molecules linked to the hydrophilic nature of chitosan [31]. A peak observed around 102.33 °C may be related to rearrangement of chitosan polymer chains and disruption of intermolecular hydrogen bonding within the polymeric network [32]. In addition, a minor thermal transition was observed in the higher temperature region between 311.54 °C and 324.52 °C with a peak temperature at 321.00 °C, corresponding to thermal degradation and decomposition of the chitosan polymer backbone. A small exothermic transition around 281.00 °C may be associated with intermediate structural rearrangements and degradation reactions occurring before complete decomposition.

**Figure 3:**
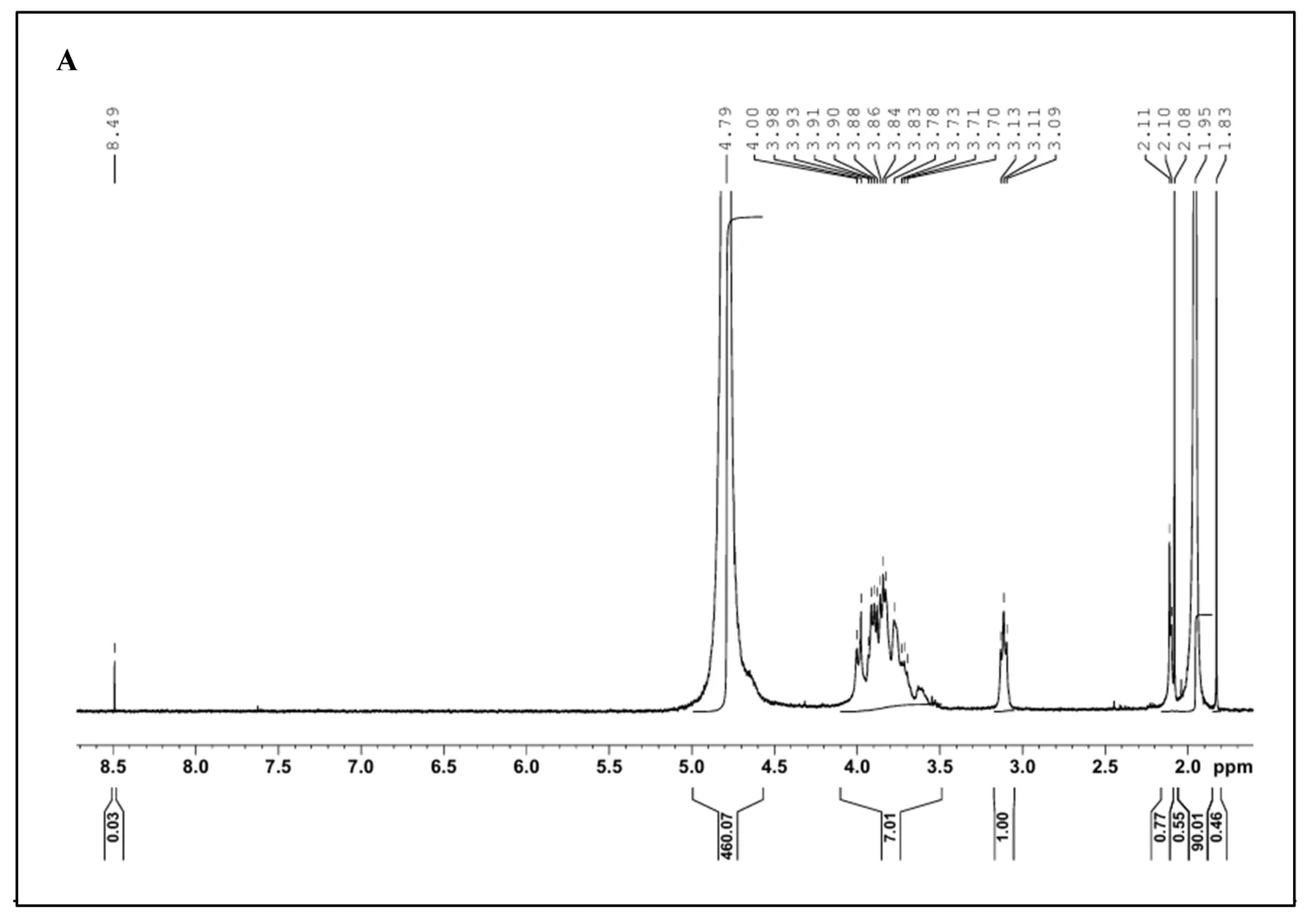

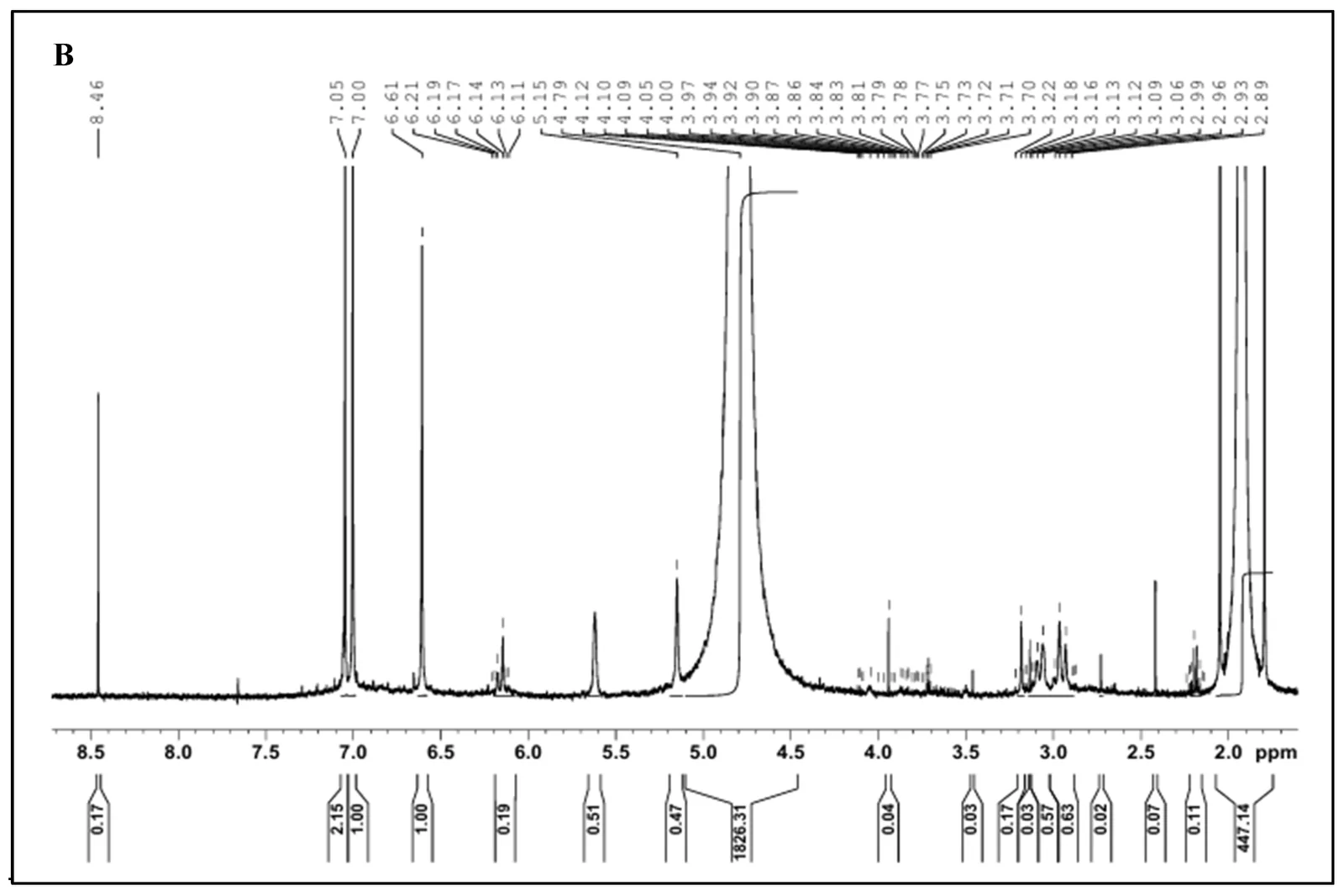
Nuclear magnetic resonance (NMR) analysis of chitosan particles (CP) (**A**) and chitosan–shrimp shell protein hydrolysate–EGCG conjugate (C–SEC) hybrid particles (**B**).

**Figure 4:**
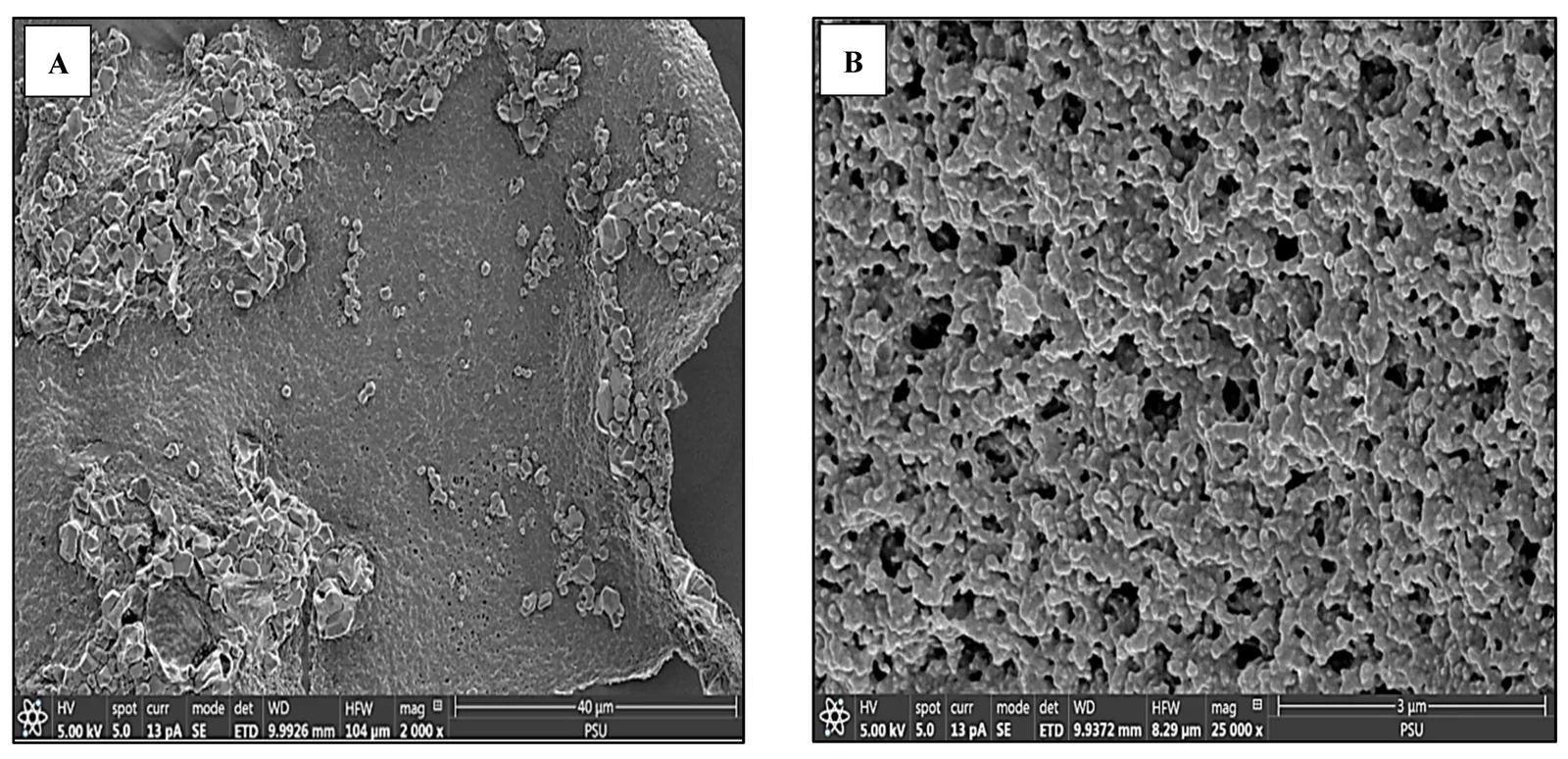

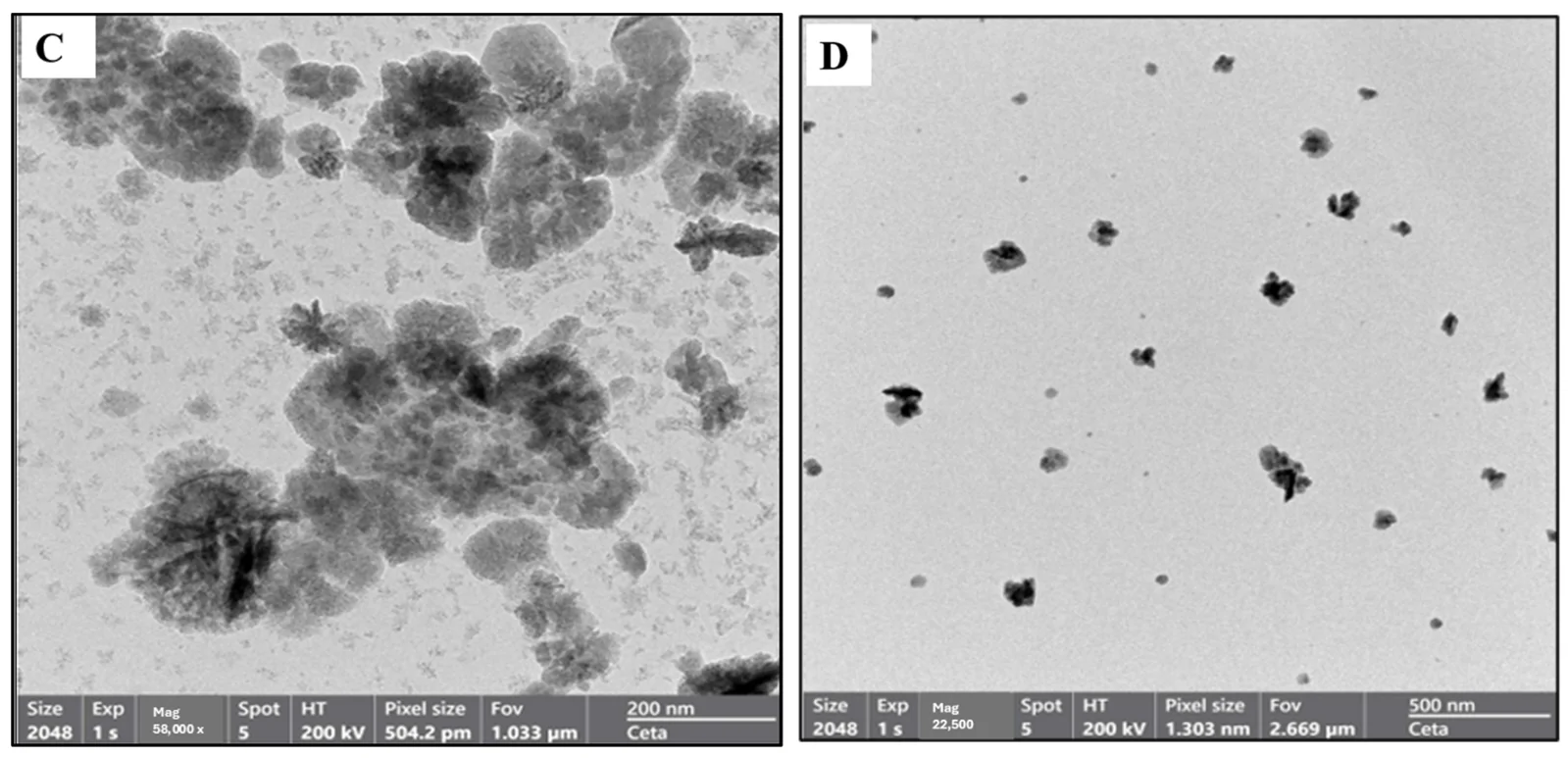
Scanning electron microscopy (SEM) (**A**,**B**) and transmission electron microscopy analysis (**C**,**D**) of chitosan particles (CP; **A**,**C**) and chitosan–shrimp shell protein hydrolysate–EGCG conjugate (C–SEC; **B**,**D**) hybrid particles. The images were obtained at different magnifications to show the morphological features of the particles, with the corresponding scale bars provided in each image.

In comparison, the C–SEC–1:5 particles showed a broad endothermic transition between 55.31 °C and 124.97 °C with a peak temperature of 60.00 °C and a substantially higher enthalpy value of 2449.60 J g^−1^. The increased enthalpy and thermal transition suggested enhanced water-binding capacity and stronger intermolecular interactions within the hybrid particle system [10]. The pronounced increase in enthalpy should not be attributed solely to enhanced water-binding capacity. Rather, the observed thermal response may reflect the combined effects of increased water–matrix interactions and changes in the molecular organization of the hybrid particle system. The presence of hydrophilic peptide residues from SPH and multiple hydroxyl groups from EGCG likely promoted hydrogen bonding interactions and stabilization of bound water molecules within the matrix [33]. A peak around 101.44 °C further indicated modification of the internal structural arrangement of chitosan following incorporation of the SEC. These structural changes may require additional energy during the thermal transition and thus contribute to the observed increase in enthalpy. The hybrid particles also exhibited a thermal decomposition peak at approximately 319.00 °C, which was relatively broader than that of CP. The broadened transition pattern indicated formation of a more heterogeneous and interconnected polymeric network due to interactions among chitosan, peptide fractions, and EGCG molecules [34]. This interpretation is further supported by the NMR peak broadening observed in Figure 3, which may indicate altered molecular mobility and/or heterogeneous molecular environments, together with the altered particle morphology observed by SEM/TEM (Figure 4). These complementary observations suggest that SEC incorporation modified the molecular and morphological organization of the chitosan matrix. However, they do not independently establish the specific contribution of molecular rearrangement to the DSC enthalpy.Similar thermal behavior was reported for whey protein isolate–chitosan polyelectrolyte hybrid particles, where interactions between chitosan and protein molecules modified the thermal transition profile and improved the thermal stability of the hybrid system [35]. This observation was consistent with the TGA results, where C–SEC–1:5 exhibited a shift of the major degradation region toward higher temperatures than CP, further supporting the formation of a more thermally stable hybrid network.

#### 3.2.4. Nuclear Magnetic Resonance (NMR)

The ^1^H NMR spectrum of CPs exhibited characteristic resonance signals corresponding to the chitosan backbone structure (Figure 3A). The signal at approximately 3.09–3.13 ppm was assigned to H-2 of the glucosamine unit, whereas the overlapping resonances between approximately 3.7 and 4.0 ppm were attributed predominantly to H-3–H-6 [36]. The intense signal around 4.7–4.8 ppm was mainly associated with residual solvent and hydroxyl proton interactions [37]. In addition, weak resonance signals observed near 2.0 ppm may correspond to methyl protons of residual N-acetyl groups present in partially deacetylated chitosan [38]. Overall, the spectrum confirmed the typical proton environment of the chitosan polymeric framework.

In contrast, the ^1^H NMR spectrum of C-SEC-1:5 exhibited substantial spectral modifications compared to CP (Figure 3B). The hybrid spectrum showed increased signal complexity with additional resonances in the aromatic region between approximately 6.1 and 7.1 ppm and extensive signal overlap in the region of approximately 2.9–4.2 ppm. These changes indicated the presence of additional proton environments contributed by the peptide and EGCG components of the SEC [39]. In particular, signals at approximately 6.13–6.21, 6.61, and 7.00–7.05 ppm were consistent with the aromatic protons of EGCG, although aromatic amino acid residues from the peptide fraction may also have contributed to the signals near 7.0 ppm. Thus, the appearance of these resonances supported the presence of the phenolic component in the C-SEC-1:5 preparation [40]. Furthermore, changes in the intensity and broadening of signals within the 3.0–4.0 ppm region indicated that incorporation of SEC altered the local chemical environment and molecular mobility of the chitosan-chain protons [41]. The observed peak broadening may reflect restricted molecular mobility, overlapping resonances, and molecular associations among chitosan, peptides, and EGCG. Additional resonance signals observed in the aliphatic region may also be associated with amino acid residues originating from the protein hydrolysate component. The modifications observed in the hybrid particle spectrum suggested that incorporation of the SEC altered the chemical environment of the chitosan matrix and promoted formation of a structurally integrated hybrid system. The absence of completely separated characteristic peaks for free EGCG or isolated peptide fractions further indicated molecular-level dispersion of the bioactive compounds within the chitosan network rather than simple physical mixing [20]. The spectral broadening and increased heterogeneity observed in the hybrid particles were consistent with the altered thermal behavior observed in TGA and DSC (Figure 1 and Figure 2).

#### 3.2.5. Scanning Electron Microscopy (SEM)

The surface morphologies of CPs and C–SEC–1:5 were examined using SEM (Figure 4A,B), respectively. Distinct morphological differences were observed following incorporation of the SEC into the chitosan matrix. The SEM image of CP exhibited a dense, compact, and continuous sheet-like morphology with a relatively smooth surface and limited porosity. Such a compact structure suggests strong intermolecular interactions among chitosan chains formed during the alkalization process [42]. In contrast, C-SEC-1:5 exhibited a markedly rougher and more porous morphology with an interconnected network containing numerous voids distributed throughout the matrix. The disappearance of the dense continuous structure observed in CP indicated that incorporation of SEC altered the self-assembly behavior of chitosan during particle formation. The peptide fractions of SPH and the multiple phenolic hydroxyl groups of EGCG likely interacted with chitosan, disrupting the strong chitosan–chitosan associations and promoting the formation of a less compact hybrid network. Similarly, Xu et al. [43] reported that self-assembly between whey protein isolate and chitosan produced composite particles with altered surface morphology due to electrostatic interactions, hydrogen bonding, and hydrophobic interactions between the two biopolymers, resulting in a more heterogeneous microstructure. The porous and heterogeneous morphology is consistent with the particle size analysis, which showed a substantial reduction in average particle size, suggesting that SEC incorporation restricted particle growth and promoted the formation of finer hybrid particles. Similar morphological changes have been reported in chitosan–protein composite systems, where intermolecular interactions between chitosan and protein components disrupted the compact polymeric structure and generated rougher, more heterogeneous morphologies [44]. Collectively, these findings indicate that incorporation of the SEC significantly altered the microstructure of chitosan, leading to the formation of a more interconnected hybrid network.

#### 3.2.6. Transmission Electron Microscopy (TEM)

TEM was performed to further examine the internal morphology and particle distribution of CP and C–SEC–1:5 (Figure 4C,D). The TEM image of CP revealed the presence of densely packed aggregates composed of fused quasi-spherical particles with irregular cluster formation. Individual particles were difficult to distinguish in several regions because of extensive particle–particle association, indicating that chitosan particles formed compact agglomerates during the alkalization process. The pronounced aggregation observed by TEM was consistent with the larger average particle size (738 nm), suggesting that extensive chitosan–chitosan interactions promoted particle growth and agglomeration. In contrast, C–SEC–1:5 exhibited substantially smaller hybrid particles that were more uniformly dispersed throughout the matrix, although small clusters containing several particles were still observed. The reduction in particle aggregation following incorporation of the SEC indicates that the peptide fractions of SPH and the polyphenolic EGCG molecules modified the self-assembly behavior of chitosan during particle formation, which resulted in the formation of a smaller particle size (112 nm) for C–SEC–1:5 than the CP. Similar reductions in particle aggregation following protein-chitosan self-assembly have been reported by Xu et al. [43], who attributed the formation of smaller and more homogeneous composite hybrid particles to intermolecular interactions between whey protein isolate and chitosan. Overall, the TEM observations corroborated the particle size analysis and provide direct visual evidence that incorporation of SEC effectively regulated particle assembly, producing smaller and more uniformly distributed hybrid particles than CP.

### 3.3. Antioxidant Potential of CP and C–SEC–1:5 in Different Oxidation Model Systems at Various Levels

#### 3.3.1. Lecithin Liposome Oxidation Model System

**Figure 5:**
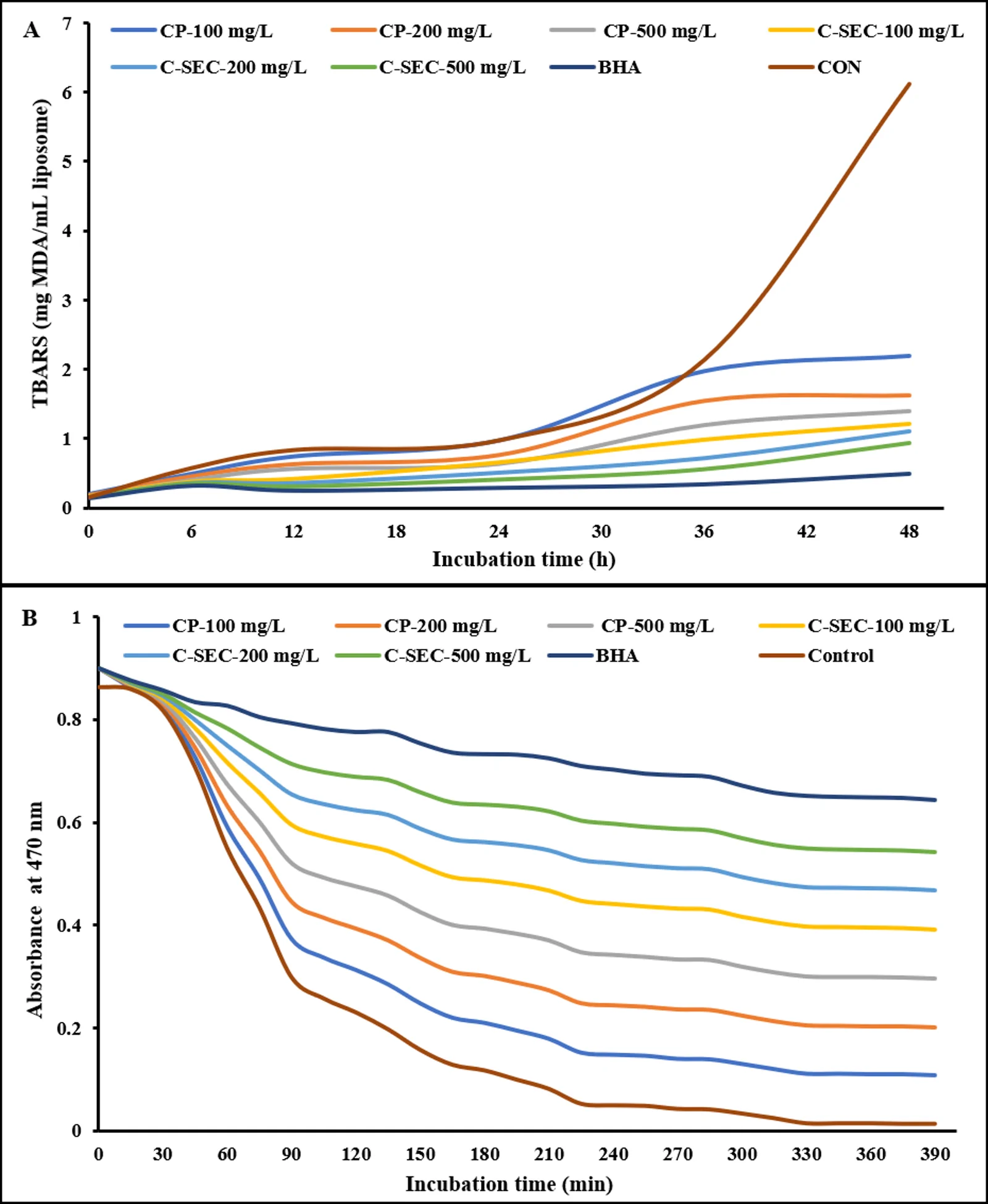
Lecithin liposome oxidation assay (**A**) and β-carotene–linoleic acid bleaching assay (**B**) of chitosan particles (CP) and chitosan–shrimp shell protein hydrolysate–EGCG conjugate (C-SEC) hybrid particles. For caption, see Table 1.

The antioxidant efficacy of CP and C–SEC–1:5 hybrid particles was evaluated using a lecithin liposome oxidation model by monitoring the formation of thiobarbituric acid reactive substances (TBARSs) during 48 h of Cu^2+^–induced oxidation (Figure 5A). TBARS values increased progressively with incubation time in all treatments, reflecting the continuous progression of lipid oxidation, in which the control (CON; without antioxidant) exhibiting the highest TBARS values throughout the incubation period. Conversely, the positive control, BHA, effectively inhibited lipid oxidation and maintained the lowest TBARS values during the entire incubation period. Both CP and C–SEC–1:5 significantly suppressed lipid oxidation compared with the CON and their inhibitory effects increased in a concentration–dependent manner (100 to 500 mg/L). Furthermore, at each tested concentration, the C–SEC–1:5 consistently exhibited significantly lower TBARS values than CP (*p* < 0.05). Among all the tested concentrations, 500 mg/L exhibited the greatest inhibition of lipid oxidation, followed by 200 mg/L and 100 mg/L. Although BHA exhibited the strongest antioxidant activity, C–SEC–1:5 at 500 mg/L achieved substantial inhibition of lipid peroxidation and represented the closest performance to the positive control among the tested particle treatments. The superior performance of C–SEC–1:5 compared with CP may be associated with the incorporation of SEC into the chitosan-based system, which provides additional antioxidant functionality.

The lower TBARS formation observed for C–SEC–1:5 indicates its ability to protect membrane lipids against oxidative degradation rather than merely scavenging free radicals in solution. The reduced TBARS formation further suggests that incorporation of SEC may have delayed both the initiation and propagation phases of lipid peroxidation, thereby suppressing the formation of secondary oxidation products during the oxidation. The improved oxidative stability may be attributed to the ability of the hybrid particles to provide sustained antioxidant protection at the lipid–water interface, where lipid oxidation is predominantly initiated [45]. The chitosan matrix likely acts as an effective carrier for the SEC, facilitating the distribution and retention of antioxidant-active components in close proximity to the phospholipid membrane [46]. This structural organization may enhance interactions between the antioxidant functional groups and lipid-derived radicals. Furthermore, the concentration-dependent reduction in TBARS formation indicates that increasing the concentration of C-SEC-1:5 enhanced the availability of antioxidant-active components, thereby providing greater protection against lipid oxidation and slowing the accumulation of oxidation products throughout the 48 h incubation period. The results obtained from the lecithin liposome oxidation assay are consistent with those of the DPPH, hydroxyl radical scavenging, ABTS, FRAP, and metal-chelating assays.

#### 3.3.2. β–Carotene–Linoleic Acid Bleaching Assay

The ability of CP and C–SEC–1:5 to inhibit lipid oxidation was assessed using the β-carotene–linoleic acid bleaching assay in an oil–in–water emulsion system (Figure 5B). In general, during incubation, thermal oxidation of linoleic acid generates lipid hydroperoxides that subsequently decompose into lipid peroxyl radicals (LOO•) [47]. These highly reactive radicals attack the conjugated double-bond structure of β–carotene, resulting in progressive discoloration and a corresponding decrease in absorbance at 470 nm. Therefore, greater retention of absorbance reflects enhanced inhibition of lipid oxidation and improved protection of β–carotene against oxidative degradation [48]. As shown in Figure 5B, all treatments exhibited a gradual decline in absorbance throughout the 390 min incubation period, indicating continuous oxidation of the emulsion system. The CON exhibited the fastest decrease in absorbance, demonstrating extensive oxidative degradation of β–carotene in the absence of antioxidant protection. In contrast, BHA effectively delayed β–carotene bleaching and maintained the highest absorbance throughout the incubation period. Both CP and C–SEC–1:5 significantly retarded β–carotene degradation compared with the CON, and their antioxidant effects increased with increasing concentration. Although BHA exhibited the greatest protection against β–carotene bleaching by consistently maintaining the highest absorbance throughout the incubation period, C–SEC–1:5 at 500 mg/L demonstrated the strongest antioxidant activity among the hybrid particles and provided substantially greater protection than the corresponding CP treatment. This finding indicates that incorporation of the SEC markedly enhanced the ability of chitosan particles to inhibit lipid oxidation in the oil-in-water emulsion system, although its efficacy remained lower than that of the synthetic antioxidant BHA. The results were in agreement with the lecithin liposome oxidation system. Normally, in the β–carotene–linoleic acid model, oxidation occurs predominantly at the oil–water interface, where lipid peroxyl radicals are continuously generated from oxidizing linoleic acid [49]. Consequently, the effectiveness of an antioxidant depends not only on its intrinsic antioxidant capacity but also on its ability to localize at the interfacial region and intercept reactive radicals before they propagate lipid oxidation or attack β–carotene [50]. The superior oxidative stability observed for C–SEC–1:5 suggests that incorporation of the SEC improved the interfacial antioxidant activity of the hybrid particles. Chitosan possesses film-forming and surface-active properties that enable adsorption onto emulsion droplets, while the incorporation of SEC may further enhance interfacial interactions through the amphiphilic nature of protein-derived peptides together with the aromatic polyphenolic structure of EGCG [51,52]. These interactions likely promote a more stable distribution of the particles around the lipid droplets, creating a protective interfacial layer that facilitates rapid interception of lipid peroxyl radicals immediately after their formation. Furthermore, the concentration-dependent inhibition of β-carotene bleaching indicates that increasing the concentration of C–SEC–1:5 enhanced the availability of antioxidant-active sites at the oil–water interface, providing more effective protection against oxidative deterioration. Improved interfacial coverage likely increased the probability of scavenging lipid-derived radicals before extensive oxidation could occur, thereby slowing the bleaching of β–carotene during prolonged incubation [49, 50, 53]. Thus, these findings demonstrate that C–SEC–1:5 effectively enhances the oxidative stability of lipid-containing systems through improved interfacial antioxidant protection.

## 4. Conclusions

Chitosan–shrimp shell protein hydrolysate–EGCG conjugate hybrid particles (C–SEC) were successfully fabricated with improved physicochemical and functional characteristics. Incorporation of the protein–polyphenol conjugate (SEC) promoted the formation of particles with enhanced antioxidant capacity, modified thermal behavior, and a porous interconnected morphology, suggesting strong intermolecular interactions within the hybrid matrix. Among the tested formulations, the C–SEC (1:5) ratio provided the most favorable balance between structural integrity and antioxidant performance, effectively suppressing lipid oxidation in both lecithin liposome and β-carotene–linoleic acid model systems. The use of SPH provides a sustainable alternative to conventional commercial proteins by converting a seafood-processing by-product into a value-added bioactive ingredient. SPH also contributes protein-derived antioxidant functionality to the hybrid system, while its conjugation with EGCG and subsequent incorporation into chitosan enables simultaneous waste valorization and the development of a multifunctional antioxidant material. These findings provide valuable insights into the design of sustainable antioxidant hybrid materials and establish a foundation for their future application as functional ingredients and bioactive delivery vehicles in food and nutraceutical formulations.

## Figures and Tables

**Table 1:** Antioxidant activities of chitosan hybrid particles prepared without and with the shrimp shell protein hydrolysate–EGCG conjugate (SEC).

	**CP**	**C–SEC–1:1**	**C–SEC–1:5**	**C–SEC–1:10**
HRSA (%)	23.7 ± 0.13 ^d^	60.19 ± 0.23 ^b^	70.29 ± 0.11 ^a^	47.28 ± 0.17 ^c^
DPPH–RSA (mmol TE/g solid)	10.47 ± 3.14 ^d^	12.88 ± 0.06 ^c^	14.55 ± 0.61 ^a^	13.18 ± 1.13 ^b^
ABTS–RSA (mmol TE/g solid)	4.93 ± 2.11 ^d^	7.28 ± 0.11 ^c^	9.80 ± 2.40 ^a^	7.97 ± 0.75 ^b^
FRAP (mmol TE/g solid)	4.61 ± 0.80 ^d^	7.65 ± 2.25 ^b^	10.57 ± 1.15 ^a^	7.05 ± 0.22 ^c^
MCA (mmol EE/g solid)	3.75 ± 1.12 ^d^	4.05 ± 1.14 ^c^	6.37 ± 0.42 ^a^	6.20 ± 0.44 ^b^

Values are expressed as mean ± SD (*n* = 3). Different lowercase superscripts in the same row indicate significant differences (*p* < 0.05). SEC: shrimp shell protein hydrolysate–EGCG conjugate; CP: chitosan particles prepared without SEC; C–SEC: chitosan and SEC hybrid particles, and the number indicates the chitosan to SEC ratio.

## Data Availability

The original contributions presented in this study are included in the article. Further inquiries can be directed to the corresponding authors.
